# Medical student experiences in prison health services and social cognitive career choice: a qualitative study

**DOI:** 10.1186/s12909-017-1109-7

**Published:** 2018-01-02

**Authors:** Ron Brooker, Wendy Hu, Jennifer Reath, Penelope Abbott

**Affiliations:** School of Medicine, Western Sydney University, Locked Bag 1797, Penrith, NSW 2751 Australia

**Keywords:** Medical education, Empathy, Health equity, Career choice, Social cognitive career theory, Prisons, Prisoners, General practice

## Abstract

**Background:**

One of the purposes of undergraduate medical education is to assist students to consider their future career paths in medicine, alongside the needs of the societies in which they will serve. Amongst the most medically underserved groups of society are people in prison and those with a history of incarceration. In this study we examined the experiences of medical students undertaking General Practice placements in a prison health service. We used the theoretical framework of the Social Cognitive Career Theory (SCCT) to explore the potential of these placements to influence the career choices of medical students.

**Methods:**

Questionnaire and interview data were collected from final year students, comprising pre and post placement questionnaire free text responses and post placement semi-structured interviews. Data were analysed using inductive thematic analysis, with reference to concepts from the SCCT Interest Model to further develop the findings.

**Results:**

Clinical education delivered in a prison setting can provide learning that includes exposure to a wide variety of physical and mental health conditions and also has the potential to stimulate career interest in an under-served area. While students identified many challenges in the work of a prison doctor, increased confidence (SCCT- Self-Efficacy) occurred through performance success within challenging consultations and growth in a professional approach to prisoners and people with a history of incarceration. Positive expectations (SCCT- Outcome Expectations) of fulfilling personal values and social justice aims and of achieving public health outcomes, and a greater awareness of work as a prison doctor, including stereotype rejection, promoted student interest in working with people in contact with the criminal justice system.

**Conclusion:**

Placements in prison health services can stimulate student interest in working with prisoners and ex-prisoners by either consolidating pre-existing interest or expanding interest into a field they had not previously considered. An important aspect of such learning is the opportunity to overcome negative preconceptions of consultations with prisoners.

## Background

Medical education over the past decade has been influenced by trends in medical workforce supply [1] and the “movement towards greater social accountability in medical education” [2] , p.1. Medical schools have been encouraged to develop programs that respond to local priority health needs and foster students with competencies and attitudes that will address health inequities [3–5]. An increasingly urgent and recognised health priority internationally [6] and in Australia [7] concerns people in custody, and those who are released back into the community [8].

### Health care for people in custody

Numerous reports emphatically confirm the vulnerability and unique health needs of this disproportionately young male, socially disadvantaged and isolated population [9–11]. People in custody often have multiple health problems, including high rates of mental illness, chronic health conditions, communicable diseases, alcohol misuse, tobacco smoking and illicit drug use, and their poor health is exacerbated by the underutilisation of health services in the community [9, 12–14]. Being in custody may be an opportunity to address their high health needs and to create societal benefits by improving health behaviours prior to re-entry into the community, such as through stabilisation of substance misuse [15].

Yet, despite these demonstrable needs, there is under-recruitment of health professionals into custodial health services. Highly qualified and experienced health professionals are needed to diagnose and manage multiple morbidities successfully in challenging prison environments [16]. Difficulties in recruitment of suitably competent doctors and nurses have been reported [17–20]. Challenges include the uninformed perceptions that “correctional physicians are inept and cannot find free-world employment” [21] or that a career in prison medicine lacks professional challenges that provide job satisfaction [22]. It is likely that career preferences are influenced by educational experiences as students [23].

### Medical education in prisons

Prison medicine programs that include training of medical students have been reported internationally [24–28]. These aim to foster social responsibility [26], promote more favourable attitudes to providing health care in prisons [24], and to reduce stigma about those who are in custody or have recently left [27, 29].

### Social cognitive career theory – A career choice conceptual framework

Throughout their medical education, students constantly review their career choices, which are complex decisions with long-lasting consequences [30, 31]. Influences on medical student career choices have been studied using the theoretical framework provided by Social Cognitive Career Theory (SCCT) [32–34]. Central to SCCT is the role of agency, which incorporates the vicarious, self-regulatory and self-reflective processes that operate in human adaptation and change [35, 36]. The theory holds that people have a capacity to develop their careers through the basic elements of self-efficacy (view of self as competent at an activity), outcome expectations (anticipation that performance of the activity will produce valued outcomes), and goals (intentions to engage in a certain activity) [33, 34]. Self-efficacy expectations and outcome expectations are seen as intertwined and interacting with contextual influences to affect students’ evolving career interests, goals and actions [34, 37]. One component of the SCCT is the ‘Interest Model’, in which the focus is on emerging interests that increase an individual’s practice activity and achievements prior to work entry. It is recognised that interests change and that exposure to “compelling learning experiences…enable (people) to rethink or expand their sense of their capabilities and the outcomes offered by different work activities” [34] , p.121. During the clinical years at university, learning experiences influence students’ evolving career interests and goals, as students refine their skills and set standards that determine their self-efficacy and outcome expectations [38]. Potent learning experiences also build an individual’s self-efficacy expectations indirectly by social persuasion and verbal encouragement from credible sources, and by assisting learners to manage negative affective reactions that produce anxiety [39, 40].

In this study we examined the learning experiences of medical students undertaking General Practice placements in prisons and the reported influence of this training on confidence in and understanding of working with people in custody and with a history of incarceration. We further examined the potential for medical student placements in prisons to influence the career choices of medical students using the SCCT Interest model as an explanatory framework.

## Methods

### Setting

The study was conducted with students an Australian undergraduate school leaver entry five year medical program. The medical school is relatively new, and promotes a social justice mission and community engaged teaching and learning approach. All students in each year cohort of approximately 120 students undertake general practice clinical attachments in both the third and final year of the course. In the final year placement, students can apply to undertake two days per week of their five week general practice placement in the state government-run prison health service [41]. These students attend health clinics in metropolitan, medium to maximum security male-only prisons. Students are supervised by General Practitioners and work with a multidisciplinary team that includes mental health, drug and alcohol and population health clinicians. Students may apply to undertake the placement or can be assigned to it.

### Data collection

All students who had undertaken the placement in 2012 and 2013 were invited to participate in the study through an email invitation from a research member not involved in their placement teaching or assessments. Firstly, they completed a pre and post placement questionnaire. Demographic information and free text responses about the placement, and knowledge and experience of health issues for people in contact with the criminal justice system from the questionnaire are considered in this paper. Secondly, students were also invited to either a face-to-face or telephone semi-structured interview within 1 month of their placement completion. The interview questions explored their experiences of the placement, their awareness and understanding of prison medicine and their views on working with people in prison or a history of incarceration. The interviews were undertaken by a researcher not directly associated with their teaching or assessment. Interviews were audio- recorded and transcribed.

### Data analysis

Free text from questionnaire responses and transcribed data from individual interviews were combined for data analysis. Data were initially coded by two researchers (RB and PA) using inductive thematic analysis with differences resolved through discussion [42]. Data collection and analysis continued until the research team agreed that saturation of the key themes had been reached. Data were not separately analysed according to whether students had volunteered or had been allocated to the placements, rather all data were analysed together. Themes were refined through iterative discussions with the wider research team using insights provided by the SCCT Interest Model. The final thematic analysis was subsequently reviewed by prison health staff involved in student teaching and by a former medical student who had participated in the prison-based general practice placement to further validate the study findings.

## Results

Of the 29 students who had undertaken the placement at the time of the study, pre and post placement questionnaires were completed by 18 students (ten female and eight male), and 17 students (ten female and seven male) participated in the semi-structured interviews. Interview durations ranged from 17 to 44 min, with an average of 27 min.

One participant was aged between 25 to 35 years while other participants were all under 25 years of age. Thirteen of the interviewed students had volunteered for the placement and four had been allocated to it. Reasons for volunteering for the placement included wanting to do something different, interest in prisoner health, social justice commitment, geographical convenience, suitable hours, and lack of interest in mainstream general practice. Two students had applied for the placement because of positive previous experience during elective terms in prison psychiatry and one had previously been a chaplain in a prison.

During their placements, students reported that they were exposed to a variety of learning opportunities, including hands on clinical practice with performance feedback, observational learning, and exposure to the culture and processes of a prison. Our findings fell into two main themes, namely learning experiences which focused on the content and the means of acquiring further knowledge, skills and attitudes; and reflections on career choices where students started looking to the future. Themes and subthemes are described in Table 1.

**Table 1:** Medical students learning experiences and reflections on future career choice

Themes	Subthemes
Learning experiences - influencing self-efficacy beliefs	Performance success
	Enhanced professional qualities
Reflecting on career choices - influencing outcome expectations	Working with prisoners and ex-prisoners
	The work of a doctor in prisons
	Considering societal benefit
	Aligning experience with personal values
	Consolidating existing career goals
	Career interest expansion

### Learning experiences - influencing self-efficacy beliefs

Students reported gains in clinical and consultation skills in keeping with their expectations of a clinical primary care placement. They also highlighted specific aspects of their learning which were enhanced because their placement was in a prison. These learning experiences were seen to increase skills and confidence broadly, but also to have influenced students’ self-efficacy beliefs regarding working with people in prison or with a history of incarceration through their own performance success and increased focus on a professional approach.

### Performance success

Students reported increased confidence in their management of complexity, including clinical issues such as substance misuse and mental health disorders, and the complex social context of some of their patients.

*If a patient comes to you with those sorts of issues, I don’t think you can just sort of sit there in shock. They’re going to expect a certain degree of understanding… you do have to have these experiences and know how to deal with different sorts of people*. (Student 12, volunteer, female).

A highlight was improved confidence in communication and consultation skills, including building rapport and practicing their skills in difficult consultations, and these skills were considered highly transferable.

*Communicating with patients who aren’t very cooperative or might not want to be there … that’s probably another learning objective, that is, practicing difficult communication, like difficult interviews with difficult patients.* (Student 8, allocated, female).

Some students had felt anxious about working with prisoners, to the point where even basic clinical interactions with prisoners were initially problematic. The opportunity to interact with people in prison decreased their negative preconceptions and allowed them to experience successful performance within consultations.

*Especially at first, it’s getting out of your comfort zone in terms of …physically touching, in some cases, when you’re doing examinations, people who you otherwise wouldn’t ever, you know, expect to have to talk to… But that was a challenge that you need to overcome so you overcome it and yeah, it was just based on I guess misconceptions and preconceptions.* (Student 8, allocated, female).

Debriefing with fellow students and supervisors was considered important given how challenging consultations in this environment could be, and several students noted a strong preference for always being accompanied by another student during this placement for mutual support.

*After we saw the patients we, sort of, debriefed. Because, some of the patients were a little bit full on, and they would also direct things towards the two of us, the two students. Like, not in a rude way but just, like, you know, they’d be asking us questions, and things like that, and, so, we just sort of had a debrief.* (Student 16, volunteer, female).

### Development of enhanced professional qualities

A professional approach to working with people in prison was seen as an important part of students’ learning. Strategies to reduce anxiety included consciously focusing on being non-judgmental and performing competently as a healthcare provider. Students reported that they felt more able to manage patients in prison in an empathic but non-emotional way through their placement exposure.

*We didn’t always look to see what people had done. It made me realise…you’re meant to just treat someone based on what they’ve got at the moment, … you don’t really have to know what crime they’ve done.* (Student 13, allocated, female).

### Reflection on career choices - influencing outcome expectations

#### Working with prisoners or ex-prisoners

Most students in this study had no prior exposure to prison health care or working with people in prison. For some, the provision of health care in prison was not something they had ever considered in the past.

*No one even thinks about the fact that people in custody get health care, it’s not something when you think of prisoners … You think of punishment and being locked up, … so I think it’s just a surprise for everyone really.* (Student 8, allocated, female).

The presence of good rapport and positive doctor-patient relationships in the prison could be unexpected observations.

*I was surprised at how the inmates interacted with the doctor. Lots of them were quite - they seemed to be quite friendly with them.* (Student 13, allocated, female).

Their prior experience with prisoners had usually been in the hospital setting where prisoners were brought in under custodial escort for emergency treatment or to see specialist doctors in outpatient clinics. Several students had witnessed the stigma prisoners can face from healthcare providers during these prior placements, and also reflected on their own negative preconceptions about prisoners.

*[In hospital] word gets around I think that this person was in gaol or whatever and then people just automatically think that they’re going to be difficult to deal with, yeah. You might not be as gentle and warm to them.* (Student 22, volunteer, female).

Students frequently noted that the placement in the prison had increased their confidence at working with people in contact with the criminal justice system,. It was also suggested that more future doctors should be exposed to teaching within prison in order to decrease the stigma faced by people with a history of incarceration.

*I think it provides a good exposure because, inevitably, you’re going to be working with someone who might have been an inmate some time during your career, and I guess I’ve gotten over any preconceptions or stereotypes that I’ve had before about working with these patients that are pretty much similar to anyone else, really.* (Student 2, volunteer, male).

#### The work of doctors in prison

Students identified the work of doctors in prisons to be different to what they had expected. Physical safety was less problematic than expected, with students of both genders reporting that custodial security processes had allayed initial apprehensions. However, while recognising security to be essential, students were surprised by how much custodial restrictions affected the work of doctors. Students commonly remarked on the need for doctors to cooperate and negotiate with the custodial team to enable care delivery.

*In a hospital, you have pretty much control of how you want to treat that patient, whereas in the correctional facility, you don’t really have that freedom… your management is restricted by the barrier of the facility and their rules.* (Student 4, volunteer, male).

Students realised that the lack of easy access to usual investigations and medications and to secondary or tertiary care required general practitioners (GPs) to manage patients with fewer resources than they would have in the community.

*I think professionally there’s more challenges because you’re so restricted … just getting basic follow up testing like a [heart ultrasound] or something that you will need a specialist to perform is a nightmare to organise.* (Student 14, volunteer, female).

The barriers to healthcare delivery were judged by several students to decrease the ability of doctors to provide best practice care. Examples given of challenges were the long waiting lists and subsequent delay in addressing potentially serious conditions, but also addressing the needs of people seen to have relatively minor problems like skin disease, and failings in continuity of care on release. For some this meant working as a doctor in prison was unappealing.

*I’d have some issues working there in regards to the conflicts between correctional and prison health services. There’s always that – for example, that delay with the patient having chest pain and he’d present three weeks later, for example. That would be one of my probably ethical problems I’d have with dealing with something like that.* (Student 3, allocated, male).

But for some students the fact that the work of a doctor in prison was different to more traditional medical practice was attractive, and was seen to offer controlled workload and hours, professional satisfaction and clinical interest.

*I found that a lot of people who worked there don’t do it for the money, but they do it more just for the whole social justice aspects… It’s stimulating in terms of both clinically reasoning and also interacting with people. I could see why people would want to do it and I think students just need to know that there’s an option.* (Student 9, volunteer, male).

Students believed observing consultations with doctors who they saw as professional role models helped their learning, such as through demonstrating how to provide respectful care and advocacy for patients within the constraints of the prison system. Positive clinical interactions by role models were sometimes contrasted with what students perceived to be examples of poor healthcare interactions in prison.

*On our first day, with one of the doctors, and we just felt awful. Like, we both kind of finished the day just being, like, “Oh my God. Like, this is an awful place to work and, you know, the system just burns you out, and you can’t really make a difference”…The second day, when we were with a different doctor who kind of had that approach of, “Yeah, we’re going to get things done for you”… it was so much more inspiring, and, like, realising that we could make a difference.* (Student 22, volunteer, female).

#### Considering societal benefits

Some students were struck by the societal benefits of effective prison health care and of ongoing care in the community for previously incarcerated people. The predominant health issues described by the students included mental health problems, chronic diseases, treatments for blood-borne viruses and drug and alcohol abuse. Students highlighted the importance of quality care for people in contact with the criminal justice system, including to limit the impact on societal health of poor health prevalent in custodial settings.

*[It is] good to get an experience with the [prison] population because eventually they will be released to the community and they will be released with all of their current co-morbidity…* (Student 14, volunteer, female).

#### Aligning experience with personal values

Several students had actively sought the placement due to their interest in promoting social equity. These students welcomed the placement as an opportunity to gain desired experience of working with underserved people and some reported that their personal values relating to social justice were reinforced and further informed by their experience. The placement enabled students to reflect on their personal values and how they would be enacted in their future career.

*When I was younger would be, like, I see suffering and I want to, like, distance myself from it, and, think that I can’t do anything… While I was in medicine, I actually, I think I realised that I don’t have to be so negative. I can actually achieve things … and, it’s also developed after [the prison placement]*. (Student 21, volunteer, female).

#### Consolidating existing career goals

Several students stated their intention was to work with underserved populations and “to make a difference”. These intentions were pre-existing and were reinforced by their learning experiences and increased self-confidence and expectations of positive outcomes of such choices.

*I chose the elective, because I was interested in forensic psychiatry, and I chose to do the GP rotation based on the fact that I had had such an enriching experience… I would be desperate to go back and work in the jails.* (Student 20, volunteer, female).

#### Career interest expansion

Some students became aware of prison health as a career option during their placement, and their understandings of this could be revelatory.

*The most interesting aspects were definitely the individuals you met through the system, also the doctors involved, just seeing how they came to that career choice and, you know, what kind of motivates them.* (Student 14, volunteer, female).

The placement experience provided some students with insights about new opportunities for practice, and stimulated interest in this previously unknown area. For these students the placement triggered reflection on their future career aspirations.

*For me it’s been really, given me some food for thought. Before I did those two placements [Prison Health and Aboriginal Health], I was pretty adamant that I wanted to go to emergency medicine, whereas now, I’m sort of weighing that up with the possibility of looking at General Practice* (Student 19, volunteer, male).

Although most students undertaking the placement did not intend to work in prisons in the future, they recognised their imminent graduation meant they would shortly be providing health care for people in contact with the criminal justice system, Students commonly noted their increased understanding would enhance their ability to work with prisoners or ex-prisoners in the future.

*I’d hope to make some sort of impact in their life by trying to have follow-up with them or trying to encourage them to seek support services… Yeah, I think I could be the best doctor you can for these sort of patients, it’s really going beyond the presenting physical illness and sort of trying to addressing all the other stuff in their life.* (Student 5, volunteer, male).

## Discussion

Our findings suggest primary care placements in prisons allow students to learn about systems of healthcare delivery, clinical skills, and being a health professional as well as initiating, consolidating or expanding the possibilities for a career in this distinctive setting. In the same way that community health experiences may foster empathy for people with health problems and reflection on practitioner responsibility to meet these needs [43], our research provides evidence that placements in prison can foster medical student awareness of the importance of working non-judgmentally and effectively with people in prison and after their release. This is important given that students acknowledged they had little prior understanding of prisoner care and often had misgivings about interactions with this population. For some, their only prior experience with prisoners had been witnessing other healthcare providers’ negative reactions and attitudes.

The SCCT framework and its Interest Model emphasises the influence of both hands-on learning experiences and vicarious learning on the career interests and goals of students. Successful practical experience directly increases self-efficacy expectations, and vicarious learning indirectly raises observers’ beliefs that they too possess the capabilities to master comparable activities [44]. In this study, the students’ increased self-efficacy and its relationship to interest in a possible career which included working with people in contact with the criminal justice system was evident. As well as substantial learning about health and healthcare systems, students reported striking gains in their confidence in communication and consultation skills in this context. This was related, importantly, to decreased apprehension in contemplating and undertaking consultations with prisoners, and contested their previously held stereotypes of people in prison. In some cases, fears were managed by sharing the experience with a colleague or a supervisor. Subsequently, these students reported greater emotional regulation by being able to recognise and manage their feelings within a framework of wellbeing, a sense of control and an ability to cope [45]. Knowing that their physical safety was protected by corrective services guards, students were able to focus on being professional and medically competent. Learning to treat all patients non-judgementally and with understanding may consolidate students’ “empathic professional” role, enabling them to display sensitivity to patients while maintaining personal respect and esteem [46].

SCCT also postulates that learning experiences build on an individual’s outcome expectations by changing their perception of the value of a career they are contemplating. Beliefs about the expected consequences of career decisions are central to the developing interests of students and their choices of future goals and actions [39]. Students who participated in this study generally perceived working with prisoners or ex-prisoners as aligned with their social justice aspirations and part of being a professional. Some also perceived the role of a doctor working in prison as potentially rewarding and interesting. While the systemic barriers to optimal care facing health professionals working in a custodial environment were confronting, they could observe prison doctors advocating for positive change. This capacity for advocacy for the vulnerable was seen by some students to mitigate against the challenges of being a prison doctor. Several students in this study reported that working in prison medicine was unappealing. Perceptions that the difficult surroundings could decrease quality of health care negatively affected their outcome expectations. Placement coordinators should thus anticipate the potential for both positive and negative student learning outcomes from such experiences and provide opportunities for debriefing and reflection.

We did not analyse the data according to whether students chose to undertake the placement or were assigned to it, due to the small number of study participants who were allocated to the term. So our findings do not illuminate whether students for such terms are more likely to have positive experiences if they select the placement. Further research may be useful to investigate if there are differential effects of training experiences in prison related to random allocation of students versus volunteer placements. Such research would need to control for different pre-existing attitudes and preconceptions at commencement, and may further inform design of effective educational interventions.

A prison health placement can reinforce the practice of empathy in medicine [47] by allowing students to witness empathic practices by experienced healthcare professionals. Contrary to the reported decline of empathy over the course of medical school [48, 49], research suggests students are supportive of doctors’ capacity to connect emotionally with patients and to have empathic attitudes towards ill people [50, 51]. Role modelling, as seen in our study, is likely to influence the retention of such personal and professional values [52]. Similarly, being aware of the stigma that people in custody or those released from prison often face when seeking medical care may allow students to address their own stereotypical views about providing health care to this underserved population [27, 28].

### Limitations

Our findings are limited to the experiences of a cohort of students at a medical school in one Australian prison service, and thus may not be transferrable to other settings given the highly contextualised nature of such learning environments. The detailed contextual description may assist readers to judge the relevance of our findings to their work settings. Participants comprised both students who volunteered and students who were allocated to the prison placement. Although not all students who volunteered for the placement did so because of a particular interest in prison health, the fact that most study participants had volunteered and several had existing interest in prison health may have led to an optimistic bias in this research. Furthermore, it may be that students with more negative views on the placement chose not to participate in this research and study participants were more likely to reflect positively on their experiences.

We used SCCT concepts as an explanatory framework to assist in interpreting findings. More research is needed to make stronger conclusions about its applicability to medical student learning in specialised settings and its potential for prediction of career choice and outcomes. The positioning of the research team members, which included clinicians working in prison settings who are educators, has influenced the design and findings from the research towards educational outcomes. However, the inclusion of a researcher (RB) who is independent of program delivery has provided a valuable and often alternative lens through which the findings have been broadened.

## Conclusion

Informed by SCCT as an explanatory framework, this study explored the learning experiences of final year medical students during a prison primary care placement. According to SCCT, interest in an activity is likely to result and endure when people view themselves as competent (self-efficacious) and they anticipate that performing the activity will produce valued outcomes (positive outcome expectations). This study provides evidence that experiential learning in the prison setting, and in particular observing the role of doctors in such settings, can lead to efficacious learning of both clinical skills and professional attributes for some students. While students identified many challenges in the work of a prison doctor, they also recognised that experience in managing people in contact with the criminal justice system was relevant to them as they entered prevocational training. Reducing anxiety associated with managing people in prison and challenging patients was important for gaining confidence in communication and consultation skills appropriate for graduate practice. A focus on empathic professionalism and having medical competence also assisted with their sense of self-efficacy. For some students, fulfilment of personal values of social equity and the value of meeting societal health needs were important outcome expectations that were reinforced by an increased understanding of the work of prison doctors.

## Abbreviations

GP: General Practitioner
SCCT: Social Cognitive Career Choice
